# Role of Molecular Targeted Therapeutic Drugs in Treatment of Oral Squamous Cell Carcinoma: Development and Current Strategies—A Review Article

**DOI:** 10.1055/s-0042-1756663

**Published:** 2022-09-19

**Authors:** Himanshu Singh, Vedant Patel

**Affiliations:** 1 Department of Oral and Maxillofacial Pathology and Oral Microbiology, Index Institute of Dental Sciences, Indore, Madhya Pradesh, India; 2 Department of Prosthodontics and Crown & Bridge, Index Institute of Dental Sciences, Indore, Madhya Pradesh, India

**Keywords:** oral squamous cell carcinoma, therapeutic drugs, targeted therapy

## Abstract

Because of active advancement in the field of biomedicine, people have in-depth knowledge of biological nature of malignant tumors and are able to recognized the overexpression of different molecules such as vascular endothelial growth factor receptor, cyclin-dependent kinase, and programmed cell death receptor. Presently, various targeted therapeutic drugs are used in different clinical trials in those patients suffering from oral squamous cell carcinoma. In this review, we converse about the various targeted therapeutic drugs and their advancement in the treatment of oral squamous cell carcinoma. This review scrutinizes the existing documentation in the literature related to the targeted therapies for oral squamous cell carcinoma. English language articles were searched in various databases such as PubMed, Scopus, Science Direct, and Google Scholar. The keywords used for searching are “oral squamous cell carcinoma,” “targeted therapy,” and “therapeutic drugs.”

## Introduction

Oral cancer is cited as tumors particularly arising in the hard palate, anterior two-thirds of the tongue, lips, upper and lower alveolar ridges, posterior deltoid muscles of molars, buccal mucosa, and oral cavity. 1 Approximately 90% of oral cancer have squamous differentiation in the mucosal epithelium; therefore, it is known as oral squamous cell carcinoma (OSCC). It is the sixth most prevalent cancer worldwide. OSCC has a survival rate of 5 years in approximately 50 to 60% of cases in the early stage. In advanced stages of OSCC, it drops to 30 to 40% of cases. Unluckily, 60 to 80% of cases of OSCC are recognized at the advanced stage. With regular development in diagnosis and treatment knowledge, the survival rate has been increased. 2 In the present scenario, proteomics, genomics, metabolomics, and different biomedical sciences have been established expeditiously. Subsequently, targeted therapies that target cancer-specific genetic targets, for example, genes responsible for invasion, division, proliferation, and metastasis of carcinogenic cells, have deliberately become the hot topic in the field of research. 3 4 Targeted therapies choose comparable therapeutic drugs as per the specific carcinogenesis location. It has the advantages of low toxicity, high selectivity, and high therapeutic indexes. In modern day, individual targeted therapeutic drugs have accomplished promising results in cancer treatment.

This review scrutinized the existing documentation in the literature related to the targeted therapies for OSCC. English language articles were searched in various databases such as PubMed, Scopus, Science Direct, and Google Scholar. The keywords used for searching are “oral squamous cell carcinoma,” “targeted therapy,” an “therapeutic drugs.”

In this review, characterization of presently most encouraging and well-known molecular targeting strategies, which is directly used in the treatment of advanced head and neck cancer, is discussed.

## Drugs Targeting the Programmed Cell Death Receptor 1

Programmed cell death receptor-1 (PD-1) is associated with the CD28 family. PD1 is expressed on natural killer cells, T-cells, B-cells, dendritic cells, and macrophages. When PD1 binds with PD-L1 (programmed cell death ligand 1), it results in apoptosis of effector T-cells, which leads to immune escape of tumor. 5 6 Also, it increases the synthesis of interleukin-10 cytokines, which suppress inflammatory responses. 7 In various studies, it is observed that overexpression of PD-L1 is seen in 50 to 90% of OSCC patients. This increased expression is positively correlated with cervical lymph node metastasis. Their coexpression was analogous to the prognosis of different malignant tumors such as melanoma and OSCC. 8 9 Presently, two drugs are used to target PD-1. They are nivolumab and pembrolizumab. These drugs are permitted for use by the U.S. Food and Drug Administration in the treatment of advanced melanoma. Pembrolizumab is used for head and neck squamous cell carcinoma patients. 10 11

## Drugs Targeting the Cyclin-Dependent Kinase (CDK) Inhibitors

The altered expression of cyclin-dependent kinases (CDK) is associated with the disproportionate proliferation of malignant cells. The cell cycle is normally regulated by cyclin and its regulatory partner, that is, CDKs. These CDKs are divided into two subgroups: cell cycle CDKs and transcriptional CDKs. The altered expression of CDKs is associated with the disproportionate proliferation of malignant cells. The cell cycle is normally regulated by cyclin and its regulatory partner, that is, CDKs. These CDKs are divided into two subgroups: cell cycle CDKs and transcriptional CDK. 12 13 The CDKs are the principal components of cell-cycle initiation and progression. In various malignancies, increased expression of cyclin and CDKs is seen. Also, decreased expression of endogenous CDK inhibitors and regulators such as CIP/KIP and INK4 is recognized in various malignancies. According to Chang et al, the CDK1 gene expression in OSCC was 17.2 times that of normal tissue. This overexpression is linked with malignant behaviors. Chen et al observed that in patients having recurrent OSCC or lymph node metastasis, CDK1 protein is overexpressed. The expression of CDK1 is considered a prognostic indicator of OSCC survival. 14 15

CDKs turn into natural targets for anticancer therapy as CDKs play a considerable role in cellular transcription and cell-cycle regulation. Various studies enlighten that CDKs inhibitors have therapeutic potential for various diseases like kidney diseases, cancer, infectious diseases, and diabetes. 16

Flavopiridol is the first CDK inhibitor that is used in human clinical trials. Flavopiridol is a semisynthetic flavonoid-based CDKs inhibitor. It is observed that flavopiridol inhibits cell proliferation by blocking G2/M and G1/S phases. Also, flavopiridol inhibits the growth of OSCC cells in a dose-dependent and time-dependent manner. Mihara et al observed that after exposure to flavopiridol, decreased expression of cyclin A, cyclin B, CDK4, CDK1, and cyclin D was seen. 17 18

## Drugs Targeting the Vascular Endothelial Growth Factor and Its Receptor Inhibitors

Tumor angiogenesis plays a pivotal role in the growth and metastasis of tumors. Hence, suppressing angiogenesis is advised to be efficient in the treatment of OSCC. The vascular endothelial growth factor (VEGF) is known as a diffusible endothelial cell-specific mitogen as well as an angiogenic factor. It is directly associated with increased vascular permeability. 19 The VEGF is an important molecule in tumor angiogenesis and is highly expressed in OSCC. Various agents that are counter to VEGF and its receptors consist of multikinase inhibitors like vandetanib and sorafenib, or monoclonal antibodies like bevacizumab. 20 21

Bevacizumab, a humanized monoclonal antibody, targets VEGF-A. It inhibits angiogenesis and increases the distribution of chemotherapeutic agents to tumor cells by reducing pressure within the tumor and by reducing microvascular permeability.

Bevacizumab inhibits biological activity that is mediated by VEGF by binding to VEGF receptors, which in turn reduces tumor angiogenesis and thereupon suppresses tumor growth. In a phase II study, it was observed that a combination of bevacizumab, cetuximab plus cisplatin was well acceptable in phase III/IVB head and neck squamous cell carcinoma, including OSCC. 22

Sorafenib, a multitargeted and multikinase inhibitor, inhibits different targets like Raf serine/threonine kinase, c-Kit, platelet-derived growth factor receptor β (PDGFR-β), and VEGFR (vascular endothelial growth factor receptor) 1–3, by inhibiting the growth and proliferation of tumor cells and also suppressing tumor angiogenesis. 23 By downregulating Mcl-1, it induces tumor cell apoptosis. Combination of sorafenib along with radiation results in synergistic effects on OSCC cells by suppressing the nuclear factor kappa B activity. 24 25

The results of preclinical trials suggested that sorafenib in consolidation with chemotherapy increases the antitumor effect by prohibiting cell growth, cell migration, and cell invasion. 26 27 Sorafenib breaks the radio-resistance of head and neck squamous cell carcinoma by prohibiting the repair of double-stranded DNA breakages. 28 29

Vandetanib, a tyrosine kinase receptor, adequately inhibits the VEGFR-2 and EGFR tyrosine kinase activities. The result of preclinical studies indicates that vandetanib inhibits the proliferation of xenograft tumor cells that includes OSCC. Vandetanib along with cisplatin and radiotherapy has the potential to conquer resistance to EGFR inhibitors during pre-clinical trials. 30

Sunitinib, a kinase inhibitor, targets the PDGFR, VEGFR, and c-Kit tyrosine kinase. It is used for the treatment of imatinib-resistant gastrointestinal stromal tumors and renal cancer. Monotherapy with sunitinib confirms unsatisfactory activity in the palliative treatment of head and neck squamous cell carcinoma. 31 32 The combination of sunitinib with cetuximab results in reduced tumor cell proliferation and increases in their differentiation. 33

## Drugs Targeting the Mammalian Target of Rapamycin Inhibitors

Mammalian Target of Rapamycin (mTOR) is a serine/threonine–protein kinase. Its function is to control cell survival, the cell cycle, and proliferation. The PI3K/AKT signal pathway shows a significant impact on the regulation of cell growth and cell proliferation. 34 As a subsequent molecule of the PI3K/AKT signal pathway, mTOR plays the principal role in the development of tumor, metastasis, invasion, and angiogenesis. In a study done by Liao et al, they observed that 85 patients out of 160 patients suffering from tongue squamous cell carcinoma showed overexpression of phosphorylates mTOR. 35 36

There are two types of mTOR inhibitors: first-generation inhibitors and second-generation inhibitors. The first-generation inhibitors were developed from rapamycin. The rapamycin forms a complex with cytoplasmic protein, that is, peptidyl-prolyl cis-trans isomerase tacrolimus binding protein. The rapamycin analogs are temsirolimus and everolimus. The second-generation mTOR inhibitors are PP242, Torin 1, and PP30. 37

Temsirolimus is an intravenous drug used for the treatment of kidney cancer. Various research studies or trials show that temsirolimus suppresses the proliferation of head and neck squamous cell carcinoma. 38

Everolimus, the derivative of rapamycin, is used as an immunosuppressant for the treatment of kidney cancer. Various studies and trials show that everolimus has antiangiogenesis and antitumor effects in the treatment of head and neck squamous cell carcinoma. 39 40

## Drugs Targeting the Epidermal Growth Factor Receptor

Epidermal growth factor receptor (EGFR), a cytoplasmic transmembrane protein, belongs to the human epidermal growth factor receptor tyrosine kinase family. It is generally made up of transmembrane domains, extracellular ligand-binding domains, and intracellular domains having tyrosinase kinase activity. 41 Various endogenous ligands are transforming growth factor-α (TGF-α), , neuregulin, and epiregulin. When these endogenous ligands are attached to the extracellular domain of EGFR, they form heterogeneous or homologous dimmers. 42 These dimmers triggered tyrosine kinases, which result in autophosphorylation of tyrosine residue and afterward triggered various downstream signaling pathways like phosphatidylinositol 3-kinase/ protein kinase B(PI3K/Akt) pathway and Ras-Raf-mitogen-activated protein kinase pathway, which give rise to antiapoptosis, proliferation, metastasis, and angiogenesis of tumor cells. 43

It is observed that higher expression of EGFR receptor is seen in well-differentiated and moderately differentiated tumors when correlated with high-grade tumors

At present, two types of drugs are used contrary to this target. These drugs are monoclonal antibodies like cetuximab and nimotuzumab, and tyrosine kinase inhibitors (TKIs) like erlotinib, gefitinib, and afatinib. 44 45

The monoclonal antibodies act by binding to the extracellular domain of the EGFR that inhibits the link between ligands and results in the inhibition of signal transmission into the cell. Cetuximab is an immunoglobulin G1 (IgG1) monoclonal antibody that is used as first-line treatment, in association with radiotherapy, for advanced head and neck squamous cell carcinoma. 46

Cetuximab can efficiently prohibit endogenous ligand-activated receptors by binding to the extracellular ligand-binding domain of EGFR, which results in increased cell apoptosis and lessened cell proliferation, metastasis, invasion, and angiogenesis.

Vermorken et al 2008 conducted a randomized phase III clinical trial in different European countries. In their study, they observed that cetuximab when combined with cisplatin extends progression-free survival (PFS) from 3.3 to 5.6 months ( *p* < 0.001), the overall survival (OS) from 7.4 to 10.1 months, and increases tumor response rate from 20 to 36% ( *p* < 0.001). 47 48

Cetuximab monotherapy for platinum-resistant recurrent or metastatic head and neck squamous cell carcinoma shows a PFS of 2.2 to 2.8 months and a response rate of 10 to 13%. 49

Nimotuzumab, a humanized IgG1 monoclonal antibody, is used in the treatment of head and neck squamous cell carcinoma, nasopharyngeal cancer, and glioblastoma. In comparison with cetuximab, it has a long half-life and moderate affinity, which substantially lowers the side effects such as skin toxicity and immunogenicity. Nimotuzumab has been directly involved in mediating antitumor effects by suppressing the survival, proliferation, and angiogenesis of cancer cells. In a study done by Xu, he observed that docetaxel–cisplatin and fluorouracil were added to nimotuzumab in the treatment of patients with advanced oral cancer, and the efficacy of the combined treatment group was 95%. In the case of the conventional chemotherapy group, the efficacy was 65%. No adverse reactions were seen in both groups. 50 These results proved that nimotuzumab in consolidation with chemoradiotherapy has extensive high value in the treatment of OSCC.

Panitumumab is a human EGFR monoclonal antibody that is used as a first-line treatment in patients with metastatic colon cancer. In a randomized phase III trial, a combination of panitumumab with chemotherapy did not show any signs of OS of patients with metastatic head and neck squamous cell carcinoma. 51 Gefitinib is the first oral EGFR-TKI. In vitro and in vivo research has concluded that it could prohibit the proliferation of oral squamous cells in a time-dependent and dose-dependent manner, which results in cell accumulation in the G1 phase, cell cycle arrest, and cell decrease in the S phase. 52 53

Erlotinib is one of the TKIs that is used in the treatment of oral cavity cancer. In vitro studies prove the effectiveness of erlotinib, in a dose-dependent manner, in the prohibition of the growth of tongue squamous cell carcinoma. 54 Erlotinib inhibits the G2/M transition and the intra-S phase of the cell cycle. Erlotinib with cisplatin and radiation shows a synergistic effect in growth inhibition of SCC-15 cells. 55

Lapatinib, a TKI, shows specificity for EGFR. In various studies, it is seen that lapatinib has an affinity for treating head and neck squamous cell carcinoma. Lapatinib with capecitabine shows effectiveness in the metastatic form of head and neck squamous cell carcinoma. 56 57

## Other Targeted Therapies

The activin receptor-like kinase 1 (ALK1) belongs to TGF-β and plays an important role in angiogenesis. Dalantercept prohibits ALK1 signaling and is an antiangiogenic agent. 58 The phase I study shows that dalantercept shows considerable ability as an anticancer therapy in head and neck squamous cell carcinoma. 59

Bortezomib, a proteasome inhibitor, is used for the treatment of mantle cell lymphoma and multiple myeloma. Primary results show a 50% control rate in patients having metastatic and recurrent head and neck squamous cell carcinoma while taking low-dose bortezomib. 60

Endostatin, a definitive endogenous angiogenesis inhibitor, inhibits the binding of VEGF to endothelial cells by adhering to integrin, heparin sulfate, and nucleolin receptor on endothelial cells, which results in suppression of tumor cell proliferation and angiogenesis. 61 Endostatin along with chemotherapy was efficient in the treatment of head and neck squamous cell carcinoma. 62

## Conclusion

Targeted therapy highlights the treatment modalities of cancer at molecular level. These therapies are extremely targeted and specific. As a likely new method, it is universally used in treatment of OSCC. It is proclaimed that in future these targeted therapies succeed the traditional methods and become choice of treatment for tumor cases.
